# Predictive role of the Mediterranean diet on mortality in individuals at low cardiovascular risk: a 12-year follow-up population-based cohort study

**DOI:** 10.1186/s12967-016-0851-7

**Published:** 2016-04-12

**Authors:** Simona Bo, Valentina Ponzo, Ilaria Goitre, Maurizio Fadda, Andrea Pezzana, Guglielmo Beccuti, Roberto Gambino, Maurizio Cassader, Laura Soldati, Fabio Broglio

**Affiliations:** Department of Medical Sciences, University of Turin, Corso Dogliotti 14, 10126 Turin, Italy; Unit of Clinical Nutrition, “Città della Salute e della Scienza” Hospital of Turin, Turin, Italy; Unit of Clinical Nutrition, Department of Internal Medicine, San Giovanni Bosco Hospital, Turin, Italy; Department of Health Sciences, University of Milan, Milan, Italy

**Keywords:** All-cause mortality, Cardiovascular risk, Cardiovascular mortality, Mediterranean diet

## Abstract

**Background:**

Adherence to the Mediterranean diet reduces the risk of all-cause and cardiovascular (CV) mortality and the incidence of CV events. However, most previous studies were performed in high-risk individuals. Our objective was to assess whether the adherence to the Mediterranean diet, evaluated by the MED score, was associated with all-cause and CV mortality and incidence of CV events in individuals at low CV risk from a population-based cohort, after a 12-year mean follow-up.

**Methods:**

A cohort of 1658 individuals completed a validated food-frequency questionnaire in 2001–2003. The MED score was calculated by a 0–9 scale. Anthropometric, laboratory measurements, and the vital status were collected at baseline and during 2014. The baseline CV risk was estimated by the Framingham risk score. Participants were divided into two groups: individuals at low risk (CV < 10) and individuals with CV risk ≥10.

**Results:**

During a 12-year mean follow-up, 220 deaths, 84 due to CV diseases, and 125 incident CV events occurred. The adherence to the Mediterranean diet was low in 768 (score 0–2), medium in 685 (score 4–5) and high in 205 (score >6) individuals. Values of BMI, waist circumference, fasting glucose and insulin significantly decreased from low to high diet adherence only in participants with CV risk ≥10. In a Cox-regression model, the hazard ratios (HRs) in low-risk individuals per unit of MED score were: HR = 0.83 (95 % CI 0.72–0.96) for all-cause mortality, HR = 0.75 (95 % CI 0.58–0.96) for CV mortality, and HR = 0.79 (95 % CI 0.65–0.97) for CV events, after multiple adjustments. In individuals with CV risk ≥10, the MED score predicted incident CV events (HR = 0.85; 95 % CI 0.72–0.99), while the associations with all-cause (HR = 1.02; 95 % CI 0.90–1.15) and CV mortality (0.94; 95 % CI 0.76–1.15) were not significant.

**Conclusions:**

Greater adherence to the Mediterranean diet was associated with reduced fatal and non fatal CV events, especially in individuals at low CV risk, thus suggesting the usefulness of promoting this nutritional pattern in particular in healthier individuals.

## Background

Findings from cohort studies have suggested that a high degree of adherence to the Mediterranean diet is associated with a reduction in all-cause and cardiovascular (CV) mortality and CV events [1–33].

The components of the Mediterranean diet have been operationalized as a dietary score using a specific and validated score: the Mediterranean diet (MED) score [7].

It is however difficult to disentangle the effects of diet from those of the risk factors for CV disease, which are increased in individuals with unhealthy diets [34–36]. Indeed, most of previous studies evaluated old subjects at high CV risk [1–6, 8, 9, 11, 15, 16, 26, 28–30, 33]. Only few studies have excluded from the analyses individuals with pre-existing CV diseases or diabetes [7, 12, 13, 32] or included younger, healthy individuals [10, 14, 16, 19, 20]. No study has explicitly considered subjects according to their CV risk.

The primary objective of this study was to assess whether the MED score was associated with all-cause mortality in individuals at low CV risk from a population-based cohort, after a 12-year mean follow-up. The secondary aim was to determine whether this score was associated with the incidence of CV events and CV mortality in the same individuals.

## Patients and methods

### Patients

All the 45–64 years old Caucasian patients (*n* = 1877) of six general practitioners were invited to participate in a metabolic screening in 2001–2003. These subjects were representative of the Local Health Units of the province of Asti (northwestern Italy) [37, 38]. Of these, 1658 (88.3 %) subjects gave their written informed consent to participate while 219 patients declined. Both the participants and non-participants were similar to the resident population of the corresponding age range in terms of male prevalence, education level, prevalence of known diabetes, and residence in a rural area [37]. The study was approved by the local ethics committee. All procedures conformed to the principles of the Helsinki Declaration.

### Methods

In the morning, after fasting, weight, height, waist circumference, and blood pressure were measured. Glucose, insulin, total cholesterol, HDL-cholesterol, triglyceride and high-sensitivity C-reactive protein (CRP) levels were determined. If the serum glucose value was ≥110 mg/dl, a second fasting glucose determination was performed. Two blood pressure measurements were made with mercury sphygmomanometers and the appropriate cuff sizes after a 10-min rest in the sitting position, and the values reported are the means of the two measurements. The waist circumference was measured by a plastic tape meter at the level of the umbilicus by trained physicians.

All participants completed the semi-quantitative food-frequency questionnaire used in the European Prospective Investigation into Cancer and Nutrition (EPIC) studies [39] and the Minnesota Leisure Time Physical Activity questionnaire [40].

From January to November 2014, patients underwent a blood sample analysis and a follow-up medical examination by their general practitioners. Information on the vital status of each patient and the causes of death of those who died was collected from the demographic files of the town of residence or death.

The laboratory methods have been described previously [37, 38]. All samples were run blindly.

### Mediterranean diet (MED) score

The validated EPIC food questionnaire assessed the average frequency and portion intake of 148 foods consumed during the 12 months prior to enrolment [39]. For each food item, the participants had to mark if the food or dish was consumed or not during the previous year. For all food items consumed, the subjects had to select their typical portion size with the help of photographs, the consumption frequency and the time period (day, week, month or year), which suited them best. Questions about the type of fat for cooking were also included. A dietician, blinded to the study details, checked all questionnaires for completeness, internal coherence, and plausibility. In case of uncertain answers, patients were interviewed by the dietician.

The reliability of the reported energy intake was assessed by calculating the ratio of estimated energy intake to predicted basal metabolic rate, using age- and sex-specific formulas derived by Schofiled [41]. Subjects with a ratio <0.88 were classified as under-reporters [42].

The MED score, assessed in accordance to Trichopoulou et al. [1, 7], included 9 components and ranged from 0 (minimum conformity) to 9 (maximum conformity). Energy-adjusted intakes at or above the sex-specific median intake for healthy foods or nutrients were given 1 point [fruits and nuts, legumes, vegetables (excluding potatoes), grains, fish, and monounsaturated fat—saturated fat ratio]; intakes lower than the sex-specific median intake for unhealthy food were given 1 point [dairy and meat] and alcohol consumption within sex-specific ranges was given 1 point (10–30 and 5–25 g/day for men and women, respectively). The original alcohol consumption proposed for men was 10–50 g/day [1, 7]; it was reduced to 10–30 g/day, in line with Italian guideline [43].

The intakes were adjusted for total energy by using the residual method [44].

The MED score was analyzed both as continuous variable (per 1-unit increase) and categorical variable: low- (0–3 units), medium- (4–5 units), and high-adherence (6–9 units).

### Definitions

The baseline CV risk score was estimated with the Framingham risk score [45]. A score <10 defined low-risk individuals.

The physical activity level was calculated as the product of the duration and frequency of each activity (in hours/week), weighted by an estimate of the metabolic equivalent of the activity and summed for the activities performed [40].

The diagnosis of CV disease was based on documented events that were recorded by the general practitioners (i.e. angina, previous myocardial infarction, coronary artery by-pass graft or another invasive procedure to treat coronary artery disease, transient ischemic attack, stroke, gangrene, amputation, vascular surgery, intermittent claudication, absent foot pulses and abnormal brachial and posterior tibial blood pressure using Doppler techniques).

The underlying cause of death was available for all the deceased patients and was obtained from the death certificate and coded according to the ICD-9 (International Classification of Diseases, Ninth Revision). Deaths due to CV diseases corresponded to ICD codes 410–414 (coronary artery diseases), 430–438 (strokes), 440 (peripheral artery diseases) and other ICD codes between 390–459 and 798.1 (other CV diseases).

### Statistical analyses

The distributions of alcohol intake, fasting insulin, triglycerides and CRP values were skewed. The characteristics of the cohort according to the MED score were analyzed by ANOVA, Kruskal–Wallis tests (for not-normally distributed variables) or the χ^2^-test, as appropriate.

To test for a linear dose–response relation, the MED score was considered as continuous variables per 1-point increment. In addition, the HR were calculated by comparing categorical variable: low, medium, and high adherence to the Mediterranean diet.

Survival data were modeled through Cox-proportional-hazards regression, by estimating the hazard ratio (HR) and its 95 % confidence intervals (CI), with successive degrees of adjustments. The model 1 was adjusted for age, sex, BMI as continuous variables; the model 2 was additionally adjusted for actual smoking (1 = yes; 0 = no) and values of systolic and diastolic blood pressure, fasting glucose, total and HDL-cholesterol (continuous); the model 3 was additionally adjusted for education (3 categories: primary schools, secondary schools, university), living in a rural area (1 = yes; 0 = no), METs (h/week), and baseline CV score (continuous variable).

Separate analyses were performed for total mortality, CV mortality and incidence of CV events. In all these analyses, individuals in the first category of MED score (low adherence) were considered as the reference group, and the other groups were introduced as dummy variables.

Sensitivity analyses in order to minimize the possibility that diet was changed in response to morbidity were also ran after excluding individuals with CV disease or diabetes at baseline. Finally, analyses were repeated after the exclusion of under-reporters.

## Results

The characteristics of individuals with CV risk <10 and ≥10 were quite different (Table 1).

**Table 1:** Clinical and laboratory characteristics of the cohort by baseline CV risk score

	CV risk score <10	CV risk score ≥10	p
Number	897	761	
Age (years)	52.0 ± 5.0	57.6 ± 4.8	<0.001
Males (%)	57.6	34.6	<0.001
Living in a rural area (%)	42.4	38.0	0.07
University (%)	9.9	4.6	
Secondary school (%)	21.3	13.9	
Primary school (%)	68.8	81.5	<0.001
Current smokers (%)	14.3	35.1	<0.001
METS (h/week)*	22.2 ± 10.3	20.6 ± 10.4	0.002
BMI (kg/m^2^)	25.8 ± 4.4	27.5 ± 4.7	<0.001
Waist circumference (cm)	89.4 ± 12.5	93.7 ± 13.2	<0.001
Systolic blood pressure (mmHg)	127.5 ± 13.0	141.0 ± 15.9	<0.001
Diastolic blood pressure (mmHg)	81.1 ± 8.6	85.9 ± 9.5	<0.001
Fasting glucose (mg/dl)	99.4 ± 16.7	111.4 ± 40.4	<0.001
Fasting insulin (µU/ml)**	8.1 ± 4.4	9.3 ± 5.4	<0.001
Total cholesterol (mg/dl)	203.9 ± 36.0	232.3 ± 40.3	<0.001
HDL cholesterol (mg/dl)	62.1 ± 14.2	58.4 ± 12.1	<0.001
Triglycerides (mg/dl)**	122.1 ± 69.8	159.4 ± 109.7	<0.001
CRP (mg/l)**	2.2 ± 5.0	3.2 ± 6.4	<0.001
CV risk score	6.3 ± 2.0	17.1 ± 7.3	<0.001
CV diseases at baseline (%)	0	12.0	<0.001
Statin use (%)	2.0	6.6	<0.001
Aspirin use (%)	1.0	10.5	<0.001

Mean ± SD

p values obtained by ANOVA or the χ^2^-test, as appropriate

*CV* cardiovascular, *CRP* C-reactive protein

* Metabolic equivalent of activity (h/week)

** p values obtained by the Kruskal–Wallis test

During a mean follow-up of 12-year, 220 individuals died, 84 of deaths were due to CV causes, and 125 new CV events were recorded by the general practitioners. Overall, individuals who died during follow-up, showed a worse metabolic pattern at baseline: they were more frequently male and showed a significantly higher age, BMI, waist circumference, fasting glucose, total cholesterol and triglycerides and lower HDL cholesterol (data not shown).

Mean and median MED score values were 3.7 and 4, respectively. Out of all participants, 768 (46.3 %) showed a low adherence, 685 (41.3 %) a medium adherence, and 205 (12.4 %) a high adherence to the Mediterranean diet (Table 2). The number of under-reporters was 138 (8.3 %). Among the three groups of adherence to the Mediterranean diet, the percentage of under-reporters did not differ (8.1, 8.7 and 8.4 % in the low-, medium-, and high-adherent groups, respectively).

**Table 2:** Characteristics of the cohort by CV risk and MED score group

	0–3	4–5	≥6	p	0–3	4–5	≥6	p
Number	406	379	112		362	306	93	
Age (years)	51.9 ± 5.4	52.0 ± 4.6	52.1 ± 4.7	0.96	57.7 ± 4.9	57.5 ± 4.8	57.7 ± 4.4	0.81
Males (%)	56.9	58.3	58.0	0.92	36.5	36.9	19.4	0.004
Living in a rural area (%)	44.6	41.4	37.5	0.36	40.3	34.3	40.9	0.23
University (%)	7.6	10.8	15.2		4.4	4.6	5.4	
Secondary school (%)	21.9	20.8	20.5		13.3	14.4	15.1	
Primary school (%)	70.4	68.3	64.3	0.18	82.3	81.1	79.6	0.98
Current smokers (%)	15.8	11.9	17.0	0.20	30.2	40.9	35.5	0.02
METS (h/week)^*^	22.7 ± 11.2	21.6 ± 9.7	22.1 ± 10.2	0.34	20.6 ± 10.5	20.7 ± 10.1	20.0 ± 10.1	0.87
BMI (kg/m^2^)	26.0 ± 4.6	25.7 ± 4.3	25.2 ± 4.2	0.22	27.8 ± 4.7	27.8 ± 4.9	25.6 ± 3.8	<0.001
Waist circumference (cm)	89.7 ± 12.6	89.4 ± 12.6	88.0 ± 11.9	0.46	94.9 ± 12.6	92.2 ± 13.4	87.1 ± 12.7	<0.001
Systolic blood pressure (mmHg)	127.6 ± 13.4	127.0 ± 12.5	128.8 ± 13.3	0.45	141.1 ± 16.2	141.2 ± 15.9	139.9 ± 14.6	0.77
Diastolic blood pressure (mmHg)	81.8 ± 8.5	80.1 ± 8.4	81.6 ± 9.4	0.01	86.1 ± 9.5	85.8 ± 9.6	85.2 ± 8.8	0.66
Fasting glucose (mg/dl)	99.5 ± 16.1	99.9 ± 18.7	97.3 ± 11.0	0.35	120.0 ± 53.0	105.7 ± 23.0	97.3 ± 11.6	<0.001
Fasting insulin (µU/ml)**	8.3 ± 5.0	7.9 ± 3.8	7.8 ± 3.2	0.43	9.7 ± 6.4	9.2 ± 4.6	7.8 ± 2.6	0.007
Total cholesterol (mg/dl)	203.0 ± 35.5	205.4 ± 36.1	202.3 ± 37.9	0.57	231.1 ± 43.8	231.2 ± 37.4	240.6 ± 34.6	0.10
HDL cholesterol (mg/dl)	62.2 ± 14.5	61.7 ± 14.0	63.1 ± 13.9	0.68	58.0 ± 11.9	58.2 ± 12.1	60.5 ± 12.6	0.18
Triglycerides (mg/dl)**	121.8 ± 71.1	124.0 ± 73.9	117.2 ± 48.2	0.81	165.9 ± 135.4	154.5 ± 82.3	150.7 ± 68.5	0.48
CRP (mg/l)**	2.1 ± 3.3	2.5 ± 6.8	1.7 ± 2.1	0.71	3.6 ± 8.3	2.9 ± 4.1	2.5 ± 2.8	0.13
CV risk score	6.3 ± 2.0	6.3 ± 2.0	6.3 ± 1.9	0.90	17.2 ± 8.1	16.9 ± 6.7	17.3 ± 6.0	0.80
Total caloric intake (kcal/day)	2169.3 ± 671.6	2058.4 ± 668.6	2126.5 ± 597.1	0.06	2022.6 ± 663.0	2025.6 ± 700.4	1964.8 ± 592.6	0.73
Vegetables (g/day)**	170.6 ± 90.6	203.3 ± 95.7	250.0 ± 144.9	<0.001	157.5 ± 90.0	195.9 ± 100.9	241.8 ± 145.9	<0.001
Legumes (g/day)**	10.8 ± 11.3	18.1 ± 14.6	21.2 ± 14.6	<0.001	14.2 ± 16.2	18.2 ± 15.7	20.9 ± 14.2	<0.001
Fruits and nuts (g/day)**	254.3 ± 157.8	412.5 ± 194.8	576.1 ± 286.1	<0.001	273.8 ± 145.9	425.2 ± 218.5	560.2 ± 154.8	<0.001
Dairy products (g/day)**	213.8 ± 185.4	169.4 ± 235.1	96.1 ± 84.0	<0.001	255.8 ± 222.4	154.2 ± 135.4	97.6 ± 78.5	<0.001
Cereals (g/day)**	209.4 ± 134.4	233.2 ± 118.0	265.5 ± 104.6	<0.001	183.5 ± 123.8	212.6 ± 94.7	257.9 ± 90.7	<0.001
Meats and meat products (g/day)	170.1 ± 116.7	138.3 ± 64.1	118.1 ± 49.1	<0.001	143.8 ± 72.1	139.1 ± 76.6	112.6 ± 46.8	<0.001
Fish (g/day)**	24.5 ± 21.3	38.1 ± 21.9	55.3 ± 35.1	<0.001	24.8 ± 20.1	38.4 ± 25.8	52.3 ± 34.9	<0.001
Monounsaturated: saturated fat ratio	1.4 ± 0.3	1.6 ± 0.3	1.9 ± 0.3	<0.001	1.4 ± 0.3	1.6 ± 0.3	2.0 ± 0.3	<0.001
Alcohol (g/day)**	21.1 ± 25.0	17.5 ± 20.6	17.4 ± 18.5	0.31	16.4 ± 24.8	16.5 ± 22.6	12.3 ± 11.8	0.32

Mean ± SD

p values obtained by ANOVA or the χ^2^-test, as appropriate

*CV* cardiovascular, *CRP* C-reactive protein

* Metabolic equivalent of activity (h/week)

** p values obtained by the Kruskal–Wallis test

In both groups (CV risk <10 and ≥10), the dietary pattern improved with increased adherence to the Mediterranean diet, as expected (Table 2). Values of BMI, waist circumference, fasting glucose and insulin significantly decreased from low to high diet adherence only in participants with CV risk ≥10 (Table 2).

Modeled as a continuous variable, the MED score predicted incident CV events in individuals with CV risk <10 and ≥10, while it was inversely associated with all-cause and CV mortality in the low-risk group only (Table 3). When the MED score was categorically modeled, the number of events decreased linearly with increasing adherence to the Mediterranean diet (Table 4). Because of the presence of few events in the categories 4–5 and 6–9, the 95 % CI were large.

**Table 3:** Association of MED score as continuous variable with outcomes by Cox-regression models

MED score	All			CV risk score <10			CV risk score ≥10		
Incident CV events									
Number	125			47			78		

	HR	95 % CI	p	HR	95 % CI	p	HR	95 % CI	p
Crude	0.83	0.73–0.93	0.002	0.80	0.66–0.98	0.03	0.85	0.73–0.99	0.04
Model 1	0.84	0.74–0.94	0.005	0.80	0.66–0.98	0.03	0.86	0.74–1.01	0.06
Model 2	0.83	0.74–0.94	0.004	0.80	0.65–0.99	0.04	0.85	0.72–0.99	0.04
Model 3	0.84	0.74–0.95	0.005	0.79	0.65–0.97	0.03	0.85	0.72–0.99	0.04
CV mortality									
Number	84			34			50		

	HR	95 % CI	p	HR	95 % CI	p	HR	95 % CI	p
Crude	0.83	0.71–0.96	0.01	0.75	0.59–0.95	0.02	0.89	0.73–1.08	0.24
Model 1	0.86	0.73–0.99	0.04	0.76	0.59–0.96	0.02	0.95	0.78–1.16	0.61
Model 2	0.87	0.75–1.02	0.09	0.74	0.58–0.95	0.02	0.96	0.78–1.18	0.70
Model 3	0.88	0.75–1.02	0.09	0.75	0.58–0.96	0.02	0.94	0.76–1.15	0.53
All-cause mortality									
Number	220			90			130		

	HR	95 % CI	p	HR	95 % CI	p	HR	95 % CI	p
Crude	0.91	0.83–0.99	0.03	0.83	0.72–0.96	0.01	0.97	0.86–1.09	0.56
Model 1	0.93	0.85–1.02	0.12	0.84	0.72–0.97	0.01	1.02	0.91–1.16	0.71
Model 2	0.94	0.85–1.10	0.17	0.84	0.72–0.97	0.02	1.02	0.90–1.15	0.75
Model 3	0.94	0.85–1.03	0.20	0.83	0.72–0.96	0.01	1.02	0.90–1.15	0.81

Model 1: adjusted for age, sex, BMI

Model 2: adjusted for age, sex, BMI, smoking habits, systolic and diastolic blood pressure, fasting glucose, total and HDL-cholesterol

Model 3: adjusted for age, sex, BMI, smoking habits, systolic and diastolic blood pressure, fasting glucose, total and HDL-cholesterol, rural area, exercise level, baseline CV risk score and education

**Table 4:** Association of MED score as categorized variable with outcomes by Cox-regression models

	Mediterranean diet score		
All	Score 0–3	Score 4–5	Score 6–9
Number	768	685	205
Incident CV events	72	42	11

	HR	HR; 95 % CI; p	HR; 95 % CI; p
Crude	1	0.65; 0.44–0.95; 0.03	0.57; 0.30–1.07; 0.08
Model 1	1	0.68; 0.46–0.99; 0.05	0.60; 0.32–1.14; 0.12
Model 2	1	0.67; 0.45–0.98; 0.04	0.58; 0.31–1.11; 0.10
Model 3	1	0.68; 0.46–1.00; 0.05	0.59; 0.31–1.12; 0.11
CV mortality	49	28	7

	HR	HR; 95 % CI; p	HR; 95 % CI; p
Crude	1	0.63; 0.40–1.00; 0.05	0.52; 0.24–1.16; 0.11
Model 1	1	0.65; 0.41–1.03; 0.07	0.65; 0.30–1.45; 0.30
Model 2	1	0.68; 0.42–1.09; 0.11	0.69; 0.31–1.54; 0.37
Model 3	1	0.68; 0.42–1.09; 0.11	0.69; 0.31–1.54; 0.37
All-cause mortality	116	82	22

	HR	HR; 95 % CI; p	HR; 95 % CI; p
Crude	1	0.78; 0.59–1.03; 0.08	0.70; 0.44–1.10; 0.12
Model 1	1	0.79; 0.59–1.05; 0.10	0.83; 0.52–1.31; 0.42
Model 2	1	0.79; 0.59–1.05; 0.11	0.84; 0.53–1.34; 0.47
Model 3	1	0.80; 0.60–1.06; 0.12	0.85; 0.54–1.35; 0.50
CV risk score <10			
Number	406	379	112
Incident CV events	28	16	3

	HR	HR; 95 % CI; p	HR; 95 % CI; p
Crude	1	0.61; 0.33–1.12; 0.11	0.38; 0.12–1.26; 0.11
Model 1	1	0.62; 0.34–1.15; 0.13	0.40; 0.12–1.31; 0.13
Model 2	1	0.62; 0.33–1.16; 0.14	0.41; 0.12–1.35; 0.14
Model 3	1	0.61; 0.33–1.14; 0.12	0.38; 0.11–1.27; 0.12
CV mortality	21	10	3

	HR	HR; 95 % CI; p	HR; 95 % CI; p
Crude	1	0.50; 0.24–1.07; 0.07	0.51; 0.15–1.71; 0.28
Model 1	1	0.52; 0.24–1.11; 0.09	0.55; 0.16–1.86; 0.34
Model 2	1	0.45; 0.21–0.97; 0.04	0.60; 0.18–2.02; 0.41
Model 3	1	0.46; 0.21–1.01; 0.05	0.59; 0.17–2.01; 0.40
All-cause mortality	50	31	9

	HR	HR; 95 % CI; p	HR; 95 % CI; p
Crude	1	0.65; 0.42–1.02; 0.06	0.64; 0.31–1.30; 0.22
Model 1	1	0.66; 0.42–1.04; 0.07	0.67; 0.33–1.37; 0.27
Model 2	1	0.65; 0.41–1.02; 0.06	0.68; 0.33–1.38; 0.28
Model 3	1	0.65; 0.41–1.03; 0.06	0.66; 0.32–1.35; 0.26
CV risk score ≥10			
Number	362	306	93
Incident CV events	44	26	8

	HR	HR; 95 % CI; p	HR; 95 % CI; p
Crude	1	0.70; 0.43–1.14; 0.14	0.71; 0.33–1.50; 0.37
Model 1	1	0.71; 0.44–1.16; 0.17	0.76; 0.35–1.63; 0.48
Model 2	1	0.69; 0.42–1.13; 0.14	0.72; 0.33–1.55; 0.40
Model 3	1	0.71; 0.43–1.16; 0.18	0.68; 0.31–1.45; 0.32
CV mortality	28	18	4

	HR	HR; 95 % CI; p	HR; 95 % CI; p
Crude	1	0.74; 0.41–1.35; 0.33	0.54; 0.19–1.54; 0.25
Model 1	1	0.73; 0.40–1.33; 0.30	0.81; 0.28–2.34; 0.70
Model 2	1	0.79; 0.43–1.44; 0.44	0.81; 0.28–2.37; 0.70
Model 3	1	0.70; 0.37–1.29; 0.26	0.76; 0.26–2.20; 0.61
All-cause mortality	66	51	13

	HR	HR; 95 % CI; p	HR; 95 % CI; p
Crude	1	0.90; 0.62–1.29; 0.56	0.74; 0.41–1.35; 0.33
Model 1	1	0.89; 0.62–1.28; 0.54	1.06; 0.58–1.94; 0.84
Model 2	1	0.90; 0.62–1.31; 0.60	1.02; 0.55–1.88; 0.95
Model 3	1	0.88; 0.61–1.29; 0.52	1.00; 0.54–1.83; 0.99

Model 1: adjusted for age, sex, BMI

Model 2: adjusted for age, sex, BMI, smoking habits, systolic and diastolic blood pressure, fasting glucose, total and HDL-cholesterol

Model 3: adjusted for age, sex, BMI, smoking habits, systolic and diastolic blood pressure, fasting glucose, total and HDL-cholesterol, rural area, exercise level, baseline CV risk score and education

Results did not significantly changed after excluding either patients with CV disease or diabetes at baseline, the under-reporters, or those on aspirin and/or on statin treatment.

## Discussion

A high adherence to the Mediterranean nutritional pattern favorably affected life expectancy and the risk of CV events and death among middle-aged individuals with a low CV risk.

The components of the Mediterranean diet are a high ratio of monounsaturated (olive oil) to saturated fat; moderate ethanol consumption (primarily wine); high consumption of legumes, cereals, vegetables, fruits and nuts; moderate/high consumption of fish; low consumption of milk and dairy products; low consumption of meat and meat products. Therefore, it is a nutritional pattern low in saturated fat, high in monounsaturated fat, complex carbohydrates and fiber and its beneficial effects on CV events and mortality may be mediated by the antioxidant, anti-inflammatory and platelet-reducing effects of its components [1–33, 46]. Furthermore, the Mediterranean diet is inversely associated with visceral adipose tissue, advanced glycation end-products and modification of circulating adipocytokines, with increase in adiponectin concentrations [26, 47–51]. Finally, intervention trials with a Mediterranean-style diet reduced values of circulating inflammatory parameters, oxidized low-density lipoproteins, markers of endothelial dysfunction [52, 53], and the incidence of CV risk factors, type 2 diabetes and the metabolic syndrome [54, 55].

However, if diabetes, hypertension, hypercholesterolemia, and chronic subclinical inflammation are potential intermediates of the association between the Mediterranean diet and CV outcomes because of the effects of the dietary pattern in modifying these risk factors, the reason why an increased MED score was more favorable in our low-risk individuals with respects to individuals with a CV risk ≥10 seems not obvious.

The diagnosis of most of these conditions may affect one’s diet with better adherence to dietary recommendations, but this seems not the case in our cohort, since mean intakes of food groups were similar among individuals with CV risk <10 and ≥10 (Table 2). Furthermore, the lack of a relevant attenuation of associations when these risk factors were adjusted for, and the absence of significant changes in the results after excluding from the analyses patients with CV diseases or diabetes at baseline did not support the possibility of mediation.

A possible explanation might be the increased influence of the higher levels of risk factors in individuals at higher risk, which might have obscured the relationships between outcomes and the MED score. Our individuals with a CV risk ≥10 were above all females and 5–6 years older than those with a CV risk <10. A more complex relationship between diet and survival with age has been reported, because of a selective earlier mortality of more susceptible individuals and relative immunity of survivors. Furthermore, an increased number of deaths occurred in our patients with a CV risk ≥10 compared to those with a CV risk <10, and an increased influence of competing causes of death may have altered the association with mortality in the former individuals. Finally, medical treatments during follow-up of the patients at higher risk could have modified these associations, thus the advancement in medical practice should be considered as a potential condition modifying survival.

Our results are in agreement with other studies which found a lower predicting role of dietary scores in older individuals [4], and stronger associations in younger and healthier individuals [7, 12–14, 16, 19, 20, 32].

It could be therefore hypothesized that a healthy diet is more important for the prevention of CV diseases than for its management. This is a highly debated topic and, contrarily to our results, other authors have shown that the association between the Mediterranean diet and the risk of death was three–five-fold higher among patients with CV diseases than healthy individuals [11]. Even if we cannot exclude the possibility that increased CV risk factors might have attenuated the relationships between outcomes and the MED score, our data clearly suggest the importance of healthy dietary pattern in low-risk individuals, since a low adherence to the Mediterranean diet was significantly associated with unfavorable events during follow-up.

The association with the MED score appeared to be stronger for mortality from CV diseases than from all-causes, albeit the CIs were wide due to the reduced number of events. This was in line with other studies finding a stronger protective role of the Mediterranean diet against CV death [7, 8, 15, 16, 25]. Indeed, among all-cause mortality, other conditions unlikely to be related to diet, such as injuries, infections, suicides, were comprised. Once we controlled for confounding, this inverse association was robust. A realistically achievable change in diet—e.g. a 1 point increment—was associated with a significant reduction in both CV mortality and CV events in individuals with a CV risk <10. Accordingly, most observational studies specifically focused on nonfatal CV events found a significantly reduced incidence of CV morbidity in individuals with a high adherence to the Mediterranean diet [6, 13, 17, 19, 22, 23, 27, 29–31]. Furthermore, our results were consistent with the protective effect found in randomized trials both in primary prevention [26] and against re-infarction in survivors of myocardial infarction [2, 56].

Several dietary factors, not included in the MED score, have been demonstrated to be important for CV risk, such as *trans* fatty acids and n-3 fatty acids. However, the omitted dietary components are likely to be associated with foods that are included in the MED score. Furthermore, the overall dietary pattern was found to be more important for health than individual nutritional components [1, 7, 9, 10] since there may be biologic interactions and complex and co-linear associations between different components of the diet and, above all, people do not consume isolated components but a complex of nutrients.

This study was performed in Northern Italy, in Piedmont, a region not facing the sea and bordering with France. This is the reason why the proportion of individuals with a high adherence to the Med score was low, differently from other areas of Italy, such as Southern Italy, where the consumptions of olive oil, fish, vegetables and legumes were higher [29, 33, 46, 57]. Nevertheless, a modest adherence to the Mediterranean diet was found to be useful in the prevention of CV events [17]. Furthermore, a higher MED score did not reflect per se a true Mediterranean diet; indeed, adherence to alternative variants of the Mediterranean diet showed beneficial effects on many outcomes [12, 13, 15, 27, 28, 30, 31], thus suggesting the possibility of adapting this dietary pattern to the geographical particularities and cultural habits of different populations.

### Limitations and strengths

Some study limitations deserve discussion. Subjects with early symptoms of CV diseases may have altered their diet and lifestyle. However, excluding cases with CV diseases at baseline and adjusting for CV risk factors did not notably alter the associations. Dietary measurement errors are almost unavoidable; however, the exclusion of under-reporting did not change the relationship between the scores and the outcomes. We only evaluated dietary pattern at baseline, and participants could have changed their diets before outcomes occurred. However, such changes would be more likely to lead to underestimation of the identified associations, as recently shown [58], and dietary patterns appear to be stable, in particular adherence to the Mediterranean diet [28]. Furthermore, the protective effects of the Mediterranean diet were maintained up to several years [2]. Residual confounding due to the healthier lifestyle of individuals more adherent to healthy diet cannot be excluded, even if we took into account most confounders in the analyses. We cannot exclude the possibility of a survival bias, which however would have rather weakened the association between lifestyle factors and survival.

The MED score is based on cohort- and sex-specific median values across respectively 9 food categories and allocates equal weights to each component of the score, thus arbitrarily assuming the same importance for every component; indeed, it was extensively used and validated in literature [1–33]. Finally, owing to the small number of observed events, our CIs of the hazard ratios were wide.

The strengths of the present cohort study were the longitudinal design with a long follow-up; the completeness of data at follow-up, which limited the possibility of selection bias; the population-based nature; the availability of information on several variables, which were introduced as possible confounders in the analyses.

## Conclusions

Efforts to promote adherence to a healthy dietary pattern as close as possible to the Mediterranean diet appear to be worthwhile in individuals at low CV risk.

## Abbreviations

BMI: body mass index
CRP: high-sensitivity C-reactive protein
CV: cardiovascular
EPIC: European Prospective Investigation into Cancer and Nutrition
MED: Mediterranean
METS: metabolic equivalent of activity

## References

[1] Trichoupolou A, Kouris-Blazos A, Wahlqvist ML, Gnardellis C, Lagiou P, Polychronopoulos E (1995). Diet and overall survival in elderly people. Br Med J.

[2] De Lorgeril M, Salen P, Martin JL, Monjaud I, Delaye J, Mamelle N (1999). Mediterranean diet, traditional risk factors, and the rate of cardiovascular complications after myocardial infarction. Circulation.

[3] Kouris-Blazos A, Gnardellis C, Wahlqvist ML, Trichopoulos D, Lukito W, Trichopoulou A (1999). Are the advantages of the Mediterranean diet transferable to other populations? A cohort study in Melbourne, Australia. Br J Nutr.

[4] Lasheras C, Fernandez S, Patterson AM (2000). Mediterranean diet and age with respect to overall survival in institutionalized, nonsmoking elderly people. Am J Clin Nutr.

[5] Haveman-Nies A, de Groot LPGM, Burema J, Cruz JAA, Osler M, van Staveren WA (2002). Dietary quality and lifestyle factors in relation to 10-year mortality in older Europeans. Am J Epidemiol.

[6] Martínez-González MA, Fernández-Jarne E, Serrano-Mártinez M, Marti A, Martinez JA, Martín-Moreno JM (2002). Mediterranean diet and reduction in the risk of a first acute myocardial infarction: an operational healthy dietary score. Eur J Nutr.

[7] Trichopoulou A, Costacou T, Bamia C, Trichopoulos D (2003). Adherence to a Mediterranean diet and survival in a Greek population. N Engl J Med.

[8] Knoops KTB, de Groot LCPGM, Kromhout D, Perria AE, Moreiras-Varela O, Menotti A (2004). Mediterranean diet, lifestyle factors, and 10-year mortality in elderly European men and women. JAMA.

[9] Trichopoulou A, Orfanos P, Norat T, Bueno-de-Mesquita B, Ocké MC, Peeters PH (2005). Modified Mediterranean diet and survival: EPIC-elderly prospective cohort study. Br Med J.

[10] Lagiou P, Trichopoulos D, Sandin S, Lagiou A, Mucci L, Wolk A (2006). Mediterranean dietary pattern and mortality among young women: a cohort study in Sweden. Br J Nutr.

[11] Trichopoulou A, Bamia C, Norat T, Overvad K, Schmidt EB, Tjønneland A (2007). Modified Mediterranean diet and survival after myocardial infarction: the EPIC-Elderly study. Eur J Epidemiol.

[12] Mitrou PN, Kipnis V, Thiébaut ACM, Reedy J, Subar AF, Wirfālt E (2007). Mediterranean dietary pattern and prediction of all-cause mortality in a US population. Arch Intern Med.

[13] Fung TF, Rexrode KM, Mantzoros CS, Manson JE, Willet WC, Hu FB (2009). Mediterranean diet and incidence of and mortality from coronary heart disease and stroke in women. Circulation.

[14] Buckland G, González CA, Agudo A, Vilardell M, Berenguer A, Amiano P (2009). Adherence to the Mediterranean diet and risk of coronary heart disease in the Spanish EPIC cohort study. Am J Epidemiol.

[15] Sjögren P, Becker W, Warensjö E, Olsson E, Byberg L, Gustafsson IB (2010). Mediterranean and carbohydrate-restricted diets and mortality among elderly men: a cohort study in Sweden. Am J Clin Nutr.

[16] Buckland G, Agudo A, Travier N, Huerta JM, Cirera L, Tormo MJ (2011). Adherence to the Mediterranean diet reduces mortality in the Spanish cohort of the European Prospective Investigation into Cancer and Nutrition (EPIC-Spain). Br J Nutr.

[17] Gardener H, Wright CB, Gu Y, Demmer RT, Boden-Albala B, Elkind MSV (2011). Mediterranean-style diet and risk of ischemic stroke, myocardial infarction, and vascular death: the Northern Manhattan Study. Am J Clin Nutr.

[18] Van den Brandt PA (2011). The impact of a Mediterranean diet and healthy lifestyle on premature mortality in men and women. Am J Clin Nutr.

[19] Agnoli C, Krogh V, Grioni S, Sieri S, Palli D, Masala G (2011). A priori-defined dietary patterns are associated with reduced risk of stroke in a large Italian cohort. J Nutr.

[20] Martínez-González MA, Guillén-Grima F, De Irala J, Ruíz-Canela M, Bes-Rastrollo M, Beunza JJ (2012). The Mediterranean diet is associated with a reduction in premature mortality among middle-aged adults. J Nutr.

[21] McNaughton SA, Bates CJ, Mishra GD (2012). Diet quality is associated with all-cause mortality in adults aged 65 years and older. J Nutr.

[22] Dills V, Katsoulis M, Lagiou P, Trichopoulos D, Naska A, Trichopoulou A (2012). Mediterranean diet and CHD: the Greek European prospective investigation into cancer and nutrition cohort. Br J Nutr.

[23] Misirli G, Benetou V, Lagiou P, Bamia C, Trichopoulos D, Trichopoulou A (2012). Relation of the traditional Mediterranean diet to cerebrovascular disease in a Mediterranean population. Am J Epidemiol.

[24] Hoevenaar-Blom MP, Nooyens ACJ, Kromhout D, Spijkerman AMW, Beulens JWJ, van der Schouw YT (2012). Mediterranean style diet and 12-year incidence of cardiovascular diseases: the EPIC-NL cohort study. PLoS One.

[25] Sofi F, Macchi C, Abbate R, Gensini GF, Casini A (2014). Mediterranean diet and health status: an updated meta-analysis and a proposal for a literature-based adherence score. Publ Health Nutr.

[26] Estruch R, Ros E, Salas-Salvadó J, Covas MI, Corella D, Arós F, Gómez-Garcia E (2013). Primary prevention of cardiovascular disease with a Mediterranean diet. NEJM.

[27] Tognon G, Lissner L, Sæbye D, Walker KZ, Heitmann BL (2014). The Mediterranean diet in relation to mortality and CVD: a Danish cohort study. Br J Nutr.

[28] Lopez-Garcia E, Rodriguez-Artalejo F, Li TY, Fung TT, Li S, Willet WC (2014). The Mediterranean-style dietary pattern and mortality among men and women with cardiovascular disease. Am J Clin Nutr.

[29] Tuttolomondo A, Casuccio A, Buttà C, Pecoraro R, Di Raimondo D, Della Corte V (2015). Mediterranean diet in patients with acute ischemic stroke: relationships between Mediterranean diet score, diagnostic subtype, and stroke severity index. Atherosclerosis.

[30] Lau KK, Wong YK, Chan YH, Li OY, Lee PYS, Yuen GG (2015). Mediterranean-style diet is associated with reduced blood pressure variability and subsequent stroke risk in patients with coronary artery disease. Am J Hyperten.

[31] Tektonidis TG, Åkesson A, Gigante B, Wolk A, Larsson SC (2015). A Mediterranean diet and risk of myocardial infarction, heart failure and stroke: a population-based cohort. Atherosclerosis.

[32] Prinelli F, Yannakoulia M, Anastasiou CA, Adorni F, Di Santo SG, Musicco M (2015). Mediterranean diet and other lifestyle factors in relation to 20-year all-cause mortality: a cohort study in an Italian population. Br J Nutr.

[33] Bonaccio M, Di Castelnuovo A, Costanzo S, Persichillo M, De Curtis A, Donati MB (2016). Adherence to the traditional Mediterranean diet and mortality in subjects with diabetes. Prospective results from the MOLI-SANI study. Eur J Prev Cardiol.

[34] Fung TT, Rimm EB, Spiegelman D, Rifai N, Tofler GH, Willett WC (2001). Association between dietary pattern and plasma biomarkers of obesity and cardiovascular disease risk. Am J Clin Nutr.

[35] Van Dam RM, Grievink L, Ocké MC, Feskens EJ (2003). Patterns of food consumption and risk factors for cardiovascular disease in the general Dutch population. Am J Clin Nutr.

[36] Berg CM, Lappas G, Strandhagen E, Wolk A, Torén K, Rosengren A (2008). Food patterns and cardiovascular disease risk factors: the Swedish INTERGENE research program. Am J Clin Nutr.

[37] Bo S, Gentile L, Ciccone G, Baldi C, Benini L, Dusio F (2005). The metabolic syndrome and high C-reactive protein: prevalence and difference by sex in a southern-European population-based cohort. Diabetes Metab Res Rev.

[38] Bo S, Durazzo M, Guidi S, Carello M, Sacerdote C, Silli B (2006). Dietary magnesium and fiber intake, inflammatory and metabolic parameters in middle-aged subjects from a population-based cohort. Am J Clin Nutr.

[39] Kroke A, Klipstein-Grobusch K, Voss S, Moseneder J, Thielecke F, Noack R (1999). Validation of a self-administered food-frequency questionnaire administered in the European Prospective Investigation into Cancer and Nutrition (EPIC) study: comparison of energy, protein, and macronutrient intakes estimated with the doubly labeled water, urinary nitrogen, and repeated 24-h dietary recall methods. Am J Clin Nutr.

[40] Taylor HL, Jacobs DR, Schucker B, Knudsen J, Leon AS, Debacker G (1978). Questionnaire for the assessment of leisure time physical activities. J Chronic Dis.

[41] Schofield WN (1985). Predicting basal metabolic rate, new standards and review of previous work. Hum Nutr Clin Nutr.

[42] Goldberg GR, Black AE, Jebb SA, Cole TJ, Murgatroyd PR, Coward WA (1991). Critical evaluation of energy intake data using fundamental principles of energy physiology: 1. Derivation of cut-off limits to identify under-recording. Eur J Clin Nutr.

[43] http://www.piramidealimentare.it/files_allegati/guida.pdf**(in Italian)**. Accessed 27 Feb 2016.

[44] Willett W, Stampfer MJ (1986). Total energy intake: implications for epidemiologic analyses. Am J Epidemiol.

[45] D’Agostino RB, Vasan RS, Pencina MJ, Wolf PA, Cobain M, Massaro JM (2008). General cardiovascular risk profile for use in primary care: the Framingham Heart Study. Circulation.

[46] Bonaccio M, Di Castelnuovo A, De Curtis A, Costanzo S, Persichillo M, Donati MB (2014). Adherence to the Mediterranean diet is associated with lower platelet and leukocyte counts: results from the Moli-sani study. Blood.

[47] Mantzoros CS, Williams CJ, Manson JE, Meigs JB, Hu FB (2006). Adherence to the Mediterranean dietary pattern is positively associated with plasma adiponectin concentrations in diabetic women. Am J Clin Nutr.

[48] Martínez-González MA, García-Arellano A, Toledo E, Salas-Salvadó J, Buil-Cosiales P, Corella D (2012). A 14-item Mediterranean diet assessment tool and obesity indexes among high-risk subjects: the PREDIMED trial. PLoS One.

[49] Steffen LM, Van Horn L, Daviglus ML, Zhou X, Reis JP, Loria CM (2014). A modified Mediterranean diet score is associated with a lower risk of incident metabolic syndrome over 25 years among young adults: the CARDIA (Coronary Artery Risk Development in Young Adults) study. Br J Nutr.

[50] Rodríguez JM, Leiva Balich L, Concha MJ, Mizón C, Bunout Barnett D, Barrera Acevedo G (2015). Reduction of serum advanced glycation end-products with a low calorie Mediterranean diet. Nutr Hosp.

[51] Bertoli S, Leone A, Vignati L, Bedogni G, Martínez-González MÁ, Bes-Rastrollo M (2015). Adherence to the Mediterranean diet is inversely associated with visceral abdominal tissue in Caucasian subjects. Clin Nutr.

[52] Esposito K, Marfella R, Ciotola M, Di Palo C, Giugliano F, Giugliano G (2004). Effect of a mediterranean-style diet on endothelial dysfunction and markers of vascular inflammation in the metabolic syndrome: a randomized trial. JAMA.

[53] Fitó M, Guxens M, Corella D, Sáez G, Estruch R, de la Torre R (2007). Effect of a traditional Mediterranean diet on lipoprotein oxidation: a randomized controlled trial. Arch Intern Med.

[54] Salas-Salvadó J, Bulló M, Babio N, Martínez-González MÁ, Ibarrola-Jurado N, Basora J (2011). Reduction in the incidence of type 2 diabetes with the Mediterranean diet: results of the PREDIMED-Reus nutrition intervention randomized trial. Diabetes Care.

[55] Estruch R, Martínez-González MA, Corella D, Salas-Salvadó J, Ruiz-Gutiérrez V, Covas MI (2006). Effects of a Mediterranean-style diet on cardiovascular risk factors: a randomized trial. Ann Intern Med.

[56] Trichopolou A, Bamia C, Trichopolou D (2005). Mediterranean diet and survival among patients with coronary heart disease in Greece. Arch Intern Med.

[57] Vannoni F, Spadea T, Frasca G, Tumino R, Demaria M, Sacerdote C (2008). Association between social class and food consumption in the Italian EPIC population. Tumori.

[58] Hoevenaar-Blom MP, Spijkerman AMW, Boshuizen HC, Boer JMA, Kromhout D, Verschuren WMM (2014). Effect of using repeated measurements of a Mediterranean style diet on the strength of the association with cardiovascular disease during 12 years: the Doetinchem Cohort Study. Eur J Nutr.

